# Nicotinamide Adenine Dinucleotide Supplementation Improves Cuprizone‐Induced Multiple Sclerosis‐Related Behavioral Changes in C57BL/6J Mice

**DOI:** 10.1002/brb3.70525

**Published:** 2025-05-05

**Authors:** Shuang Song, Ruoyi Guo, Jiangyuan Guo, Bin Li, Yusen Han, Huining Zhang, Li Guo

**Affiliations:** ^1^ Department of Neurology The Second Hospital of Hebei Medical University Shijiazhuang Hebei China; ^2^ Key Neurological Laboratory of Hebei Province Shijiazhuang Hebei China; ^3^ Key Laboratory of Clinical Neurology, Ministry of Education Hebei Medical University Shijiazhuang Hebei China

**Keywords:** behavioral changes, cuprizone, nicotinamide adenine dinucleotide

## Abstract

**Objective:**

To investigate whether nicotinamide adenine dinucleotide (NAD+) supplementation can improve behavioral changes in a cuprizone‐intoxicated mouse model.

**Methods:**

Six‐week‐old C57BL/6J mice were divided into three groups: two were fed 0.2% cuprizone chow (cuprizone and cuprizone + NAD+ groups), and the other group was fed normal rodent chow (control group) for 4 weeks. The mice in the cuprizone + NAD+ group received 250 mg/kg/day NAD+ intraperitoneally once a day, while the other mice were administered saline simultaneously. Behavioral tests for spatial memory (Morris water maze and Y maze), locomotor ability (grip test and rotarod test), depression‐like behavior (open field test and tail suspension test), and exploratory behavior (open field test) were conducted.

**Results:**

In the probe test of the Morris water maze, the cuprizone group spent a significantly smaller proportion of time in the target quadrant than the control group did (16.32% vs. 31.66%, *p* = 0.006). However, supplementation with NAD+ increased the value (28.78% vs. 16.32%, *p* = 0.023). Similarly, in the Y maze test, the cuprizone group demonstrated a notably lower ratio of effective alterations compared to the control group (0.543 vs. 0.648, *p* < 0.001), and the cuprizone + NAD+ group presented an improved ratio compared with the cuprizone group (0.613 vs. 0.543, *p* = 0.021). Compared with the control group, cuprizone toxicity resulted in a decreased time to fall (169.10 vs. 247.60 s, *p* = 0.015) in the grip test, but NAD+ supplementation mitigated this effect (261.60 vs. 169.10 s, *p* = 0.003). There were no significant differences in the immobile time among groups in both the tail suspension test and the open field test, and there were also no significant differences in center distance in the open field test.

**Conclusions:**

Direct NAD+ supplementation improves the locomotor ability and spatial memory of cuprizone‐intoxicated C57BL/6J mice. However, NAD+ supplementation does not show significant effects on depressive and exploratory behavior of experimental mice.

## Graphical abstract

Direct nicotinamide adenine dinucleotide supplementation improves the locomotor ability and spatial memory of cuprizone intoxicated C57BL/6 mice.

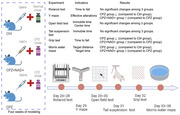

## 1 Introduction

Among patients with multiple sclerosis (MS), a significant proportion suffer from various symptoms related to behavioral changes: more than 40% suffer from gait incoordination (Yassine et al. 2024), 43%–70% suffer from cognitive impairment (Morrow 2024), 25%–65% suffer from depression, and 20%–54% suffer from anxiety (Jellinger 2024). The cuprizone‐induced demyelination model, first established in the late 1960s, is an important animal model for MS. Young adult mice (often male) were orally intoxicated with the copper chelator cuprizone (bis‐cyclohexanone oxaldihydrazone) for several weeks, after which oligodendrocyte apoptosis, innate immune cell activation, and demyelination were observed in nerve tissue (Dedoni et al. 2023). Typically, cuprizone‐intoxicated mice do not develop limb paralysis but rather subtle motor, cognitive, and anxiety‐like/depression‐like behavioral dysfunctions. Consequently, this model offers a viable platform for assessing the efficacy of interventions for MS‐related behavioral changes.

Nicotinamide adenine dinucleotide (NAD+) is a coenzyme, a cellular metabolite, and a key hydride acceptor for redox reactions, interacting with a wide range of small biological molecules (Bresque et al. 2023). Thus, the intracellular level of NAD**+** profoundly affects energy metabolism and mitochondrial function. In the experimental autoimmune encephalitis (EAE) model, NAD+ supplementation attenuated the inflammatory response in central demyelinating foci, ameliorated symptoms, reversed retinal ganglion cell apoptosis, and regulated peripheral immune cell differentiation (Guo et al. 2021; J.‐L. Wang et al. 2020; X. Wang et al. 2021). Despite the fact that direct supplementation of NAD+ has shown efficacy in the EAE model, it is important to note its limitations. NAD+ is characterized by rapid metabolism and probably limited oral bioavailability and difficulties in crossing the blood‐brain barrier (Jeje et al. 2024; Migaud et al. 2024). These factors not only restrict its effectiveness but also contribute to the relative lack of comprehensive data regarding its direct supplementation, highlighting the need for further exploration in this area. Moreover, whether direct NAD+ supplementation can influence behavioral changes in the cuprizone‐intoxicated mouse model has not been investigated.

In this study, we established a cuprizone‐intoxicated model using C57BL/6J male mice to assess the effects of NAD+ supplementation on behavioral dysregulations. As the cuprizone model effectively exhibits non‐paralytic MS‐related behavioral deficits, and direct NAD+ supplementation remains unexplored for behavioral outcomes, our study pioneers the systematic assessment of direct NAD+ administration in rescuing cuprizone‐induced MS‐related behavioral dysfunctions, thereby addressing the critical knowledge gap between preclinical model application and intervention strategy. These findings provide foundational data to optimize NAD+‐based therapeutic regimens, thereby informing targeted combinatorial therapy development for MS symptom management.

## 2 Materials and Methods

### 2.1 Animals

Six‐week‐old male C57BL/6J mice weighing approximately 16–20 g were purchased from Nanjing Junke Bioengineering Co. Ltd. The dosage and reaction of cuprizone are sex‐ and strain‐specific, and male mice are more reliable to this toxic compound (Dedoni et al. 2023). Thus, the mice used in the study were all male. The mice were fed ad libitum and housed at room temperature (24 ± 2°C) with 12‐h/12‐h light/dark cycles. The experiment was approved by the Experimental Ethics Committee of the Second Hospital of Hebei Medical University (No. 2021‐AE044).

### 2.2 Experimental Design

The mice were randomly divided into three groups (*n* = 15 for every group). At the start of the experiment, the two treatment groups (CPZ+NAD+ group and CPZ group) were fed 0.2% cuprizone (w/w) (Sigma) rodent chow (Beijing Keao Xieli Feed Co. Ltd.), and the control group (Ctrl group) was fed normal rodent chow. The CPZ+NAD+ group was administered 250 mg/kg/d NAD+ (MCE) intraperitoneally qd simultaneously at the start of the experiment along with 0.2% cuprizone rodent chow, whereas the other two groups were administered saline simultaneously at the start of the experiment along with 0.2% cuprizone or normal rodent chow. The dosage of NAD+ was determined according to previous experiments on the EAE model (Guo et al. 2021; J.‐L. Wang et al. 2020; X. Wang et al. 2021). After 4 weeks of intervention, the mice were subjected to several behavioral tests.

### 2.3 Behavioral Tests

**FIGURE 1:**
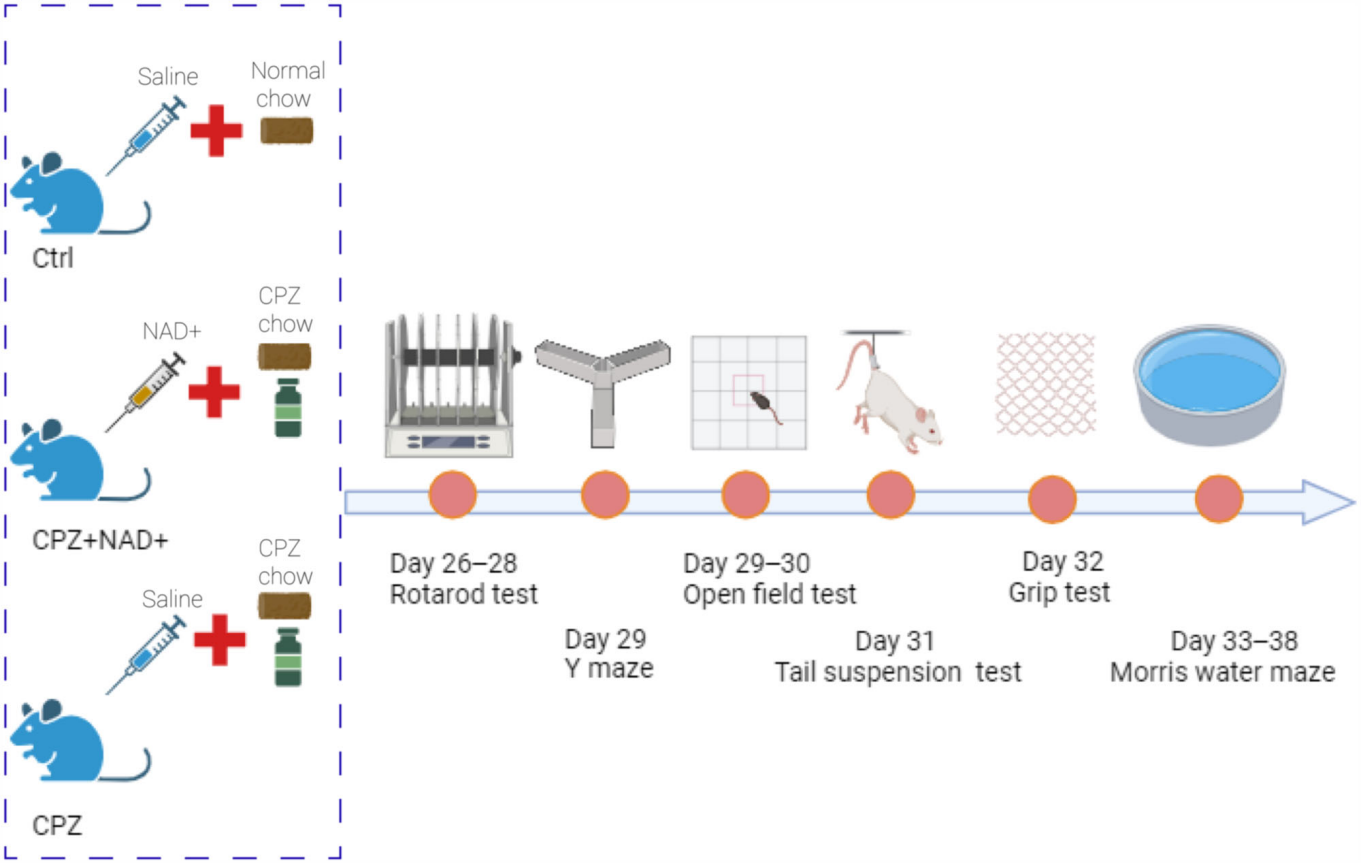
Timeline of the experiment (created in BioRender.com).

All the behavioral tests were performed during the daytime, with food and water ad libitum. All mice were subjected to the behavioral tests according to the same schedule (Figure 1).

### 2.4 Morris Water Maze

The Morris water maze (MWM) test was used to test the spatial memory of the mice (Othman et al. 2022). Briefly, the MWM tank was 140 cm in diameter and 40 cm in depth, with water (25 ± 1°C) stained with milk powder. To assist the mice in space orientation, visible markers were hung around the walls of the tanks in the MWM. An imaginary “+”‐shaped line quadripartitioned the surface of the maze water into four quadrants. The transparent, invisible round escape platform with a diameter of 12 cm was located 1 cm underneath the water. The quadrant where the platform was located was defined as the target quadrant.

The experiment was divided into two stages: a space acquisition test and a probe test. On Days 1–5, the mice underwent a space acquisition test, and they were sequentially trained to swim in the MWM and tried to find the platform within 60 s. If the mice did not find the platform on time, they were gently guided to the platform and allowed to stay for 15 s to learn. The swimming tracks were recorded by a camera and analyzed via Morris software (version 2.8.9.1, Shanghai Yishu Technology Co. Ltd.), and the time to find the platform (escape latency) was calculated.

On Day 6, the probe test was performed. The platform was removed, and every mouse was allowed to swim in the maze for 1 min. The swimming track was recorded, after which the number of platform crossings, target time% (swimming time in the target quadrant/total time × 100%), and target distance% (swimming distance in the target quadrant/total distance × 100%) were calculated.

### 2.5 Y Maze Test

The Y maze test was conducted to examine the spatial memory of the mice (Cho et al. 2024). A three‐arm wooden opaque white maze (30 cm long, 6 cm wide, and 15 cm deep, labeled A, B, or C) with a 120° angle among each other was used to test the mice. The experiment was conducted under an illuminance of ∼150 lx. Each mouse was randomly set at one end of the Y maze arm, and the number of entries into the maze arms was recorded. All mice were allowed an 8‐min session. The number of consecutive entries into the three arms was defined as an actual alteration, namely ABC, ACB, BAC, BCA, CAB, or CBA. The total number of arm alterations was the total number of arm entries minus two, which was defined as total alternations. Finally, the alteration ratio, which can reflect the cognitive function of spatial memory, was calculated as actual alterations/total alternations.

### 2.6 Rotarod Test

An apparatus containing a rod that can rotate from 4 to 40 rpm within 300 s was used (Lubrich et al. 2022). The mice were placed onto the rod, and the latency of falling down from the rod was recorded. The mice were tested three times a day for three consecutive days. The mean time to fall on every testing day and on a total of nine trials was calculated to reflect the locomotor ability of the mice.

### 2.7 Grip Test

The mice were hung on a stainless mesh, and the latency of falling down was recorded three times a day (Graber et al. 2019). The mean time duration to fall was calculated to reflect the locomotor ability of the mice.

### 2.8 Tail Suspension Test

For the tail suspension test (TST) (Lopes et al. 2020), mice were hung on a horizontal bar approximately 1/4 from the tip of the tail for 6 min, and the immobile time was recorded to reflect the depression‐like behavior of the mice.

### 2.9 Open Field Test

The open field test (OFT) was used to test the depression‐like behavior and exploratory behavior of the mice. The mice were placed in an acrylic 30 cm × 20 cm × 20 cm square box with no ceilings, and the camera was placed right above the square box. Mice were allowed a 10 min session to move freely in the square box while the moving track was recorded and analyzed by ImageJ 1.53c. The immobile time% (calculated as immobile time/total time × 100%) can reflect the depression‐like behavior of mice. The center distance% (distance in central area/total distance × 100%) and center time% (time in central area/total time × 100%) can reflect the exploratory behavior of mice (Kraeuter et al. 2019).

### 2.10 Statistical Analysis

SPSS 21.0 was used to perform the statistical analysis. The Shapiro–Wilk test was used to evaluate the normal distribution of the data. Analysis of variance (ANOVA) was used to compare normally distributed quantitative data. If the data did not follow a normal distribution, the non‐parametric Kruskal–Wallis test was used. For repeated measurement data, generalized estimating equation (GEE) was utilized. A *p* value under 0.05 was regarded as statistically significant.

## 3 Results

### 3.1 Cognitive Tests

**FIGURE 2:**
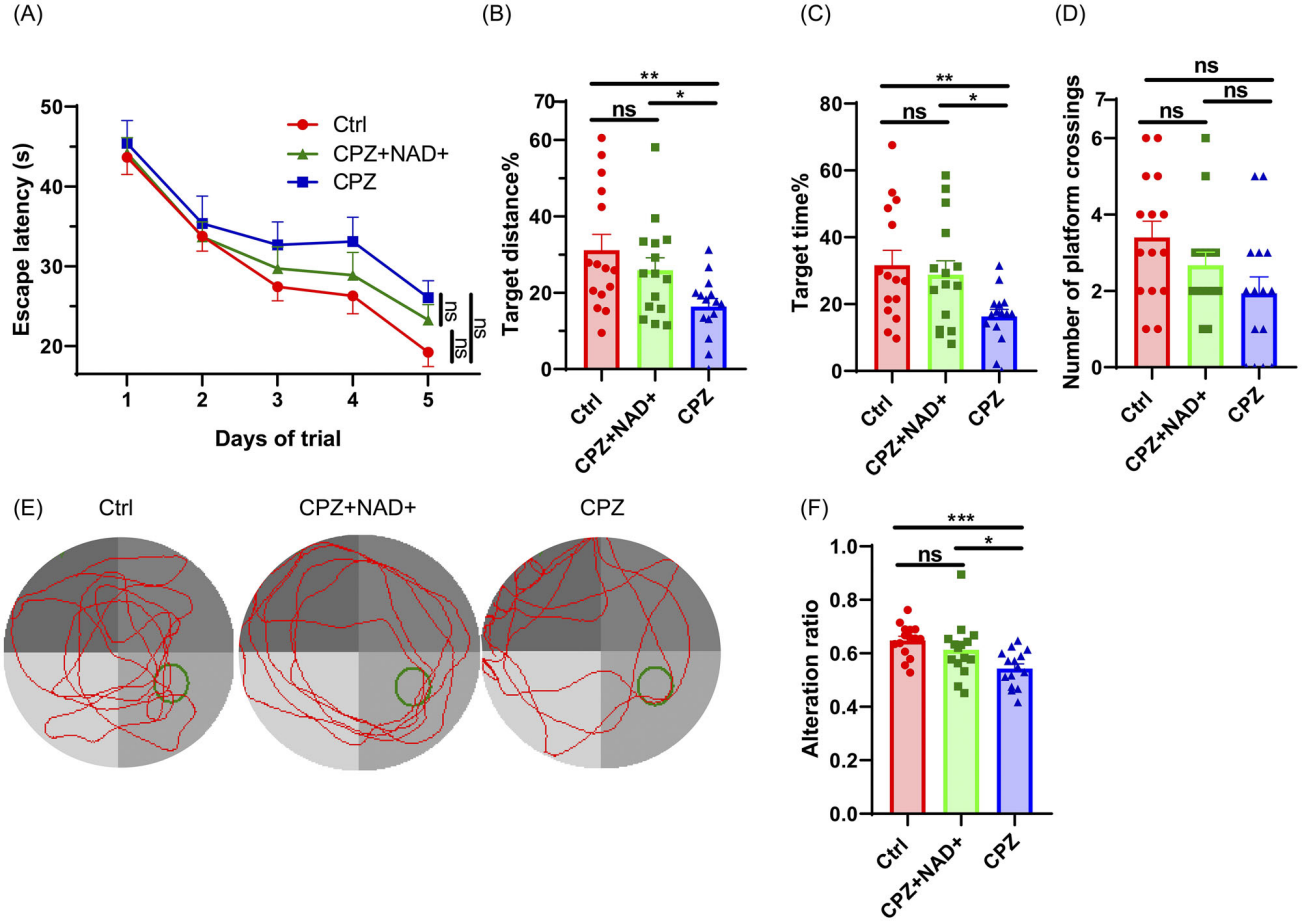
Results of MWM test and Y maze test. Data were shown as mean ± SEM. *p < 0.05, **p < 0.01, ***p < 0.001, and ns represents not significant. *N* = 15 per group. (A) Learning curve of escape latency in the space acquisition phase. (B–E) showed results of the probe phase in the MWM test. (B) Target distance% among three groups. (C) Target time% among three groups. (D) Number of platform crossings among three groups. (E) Typical swimming tracks. The green circle represents the escape platform, and the red line represents the swimming track. (F) The percentage of effective alteration among groups in the Y maze test.

In the MWM test, during the first five days of space acquisition, learning curves of escape latency did not significantly differ among groups (*p* = 0.081, Figure 2A). In the probe test, cuprizone intoxication caused a significant decrease in the target distance% compared with that in the Ctrl group (16.34% vs. 31.11%, *p* = 0.003), and NAD+ supplementation significantly increased the value compared with that in the CPZ group (25.86% vs. 16.34%, *p* = 0.046) (Figure 2B). Similarly, the target time% was significantly lower in the CPZ group than in the Ctrl group (16.32% vs. 31.66%, *p* = 0.006). The CPZ+NAD+ group had better performance than the CPZ group (28.78% vs. 16.32%, *p* = 0.023) (Figure 2C). The number of platform crossings did not reach statistical differences among groups (Figure 2D). The representative swimming tracks in the probe test are shown in Figure 2E.

In support of the MWM results, in the Y maze test, the CPZ group presented a significantly lower ratio than the Ctrl group did (0.543 vs. 0.648, *p* < 0.001), and the CPZ+NAD+ group presented an improved ratio compared with the CPZ group (0.613 vs. 0.543, *p* = 0.021) (Figure 2F).

To sum up, cuprizone intoxication impaired the spatial memory of the mice, whereas NAD+ supplementation partially ameliorated the cuprizone‐induced cognitive dysregulation.

### 3.2 Motor Tests

**FIGURE 3:**
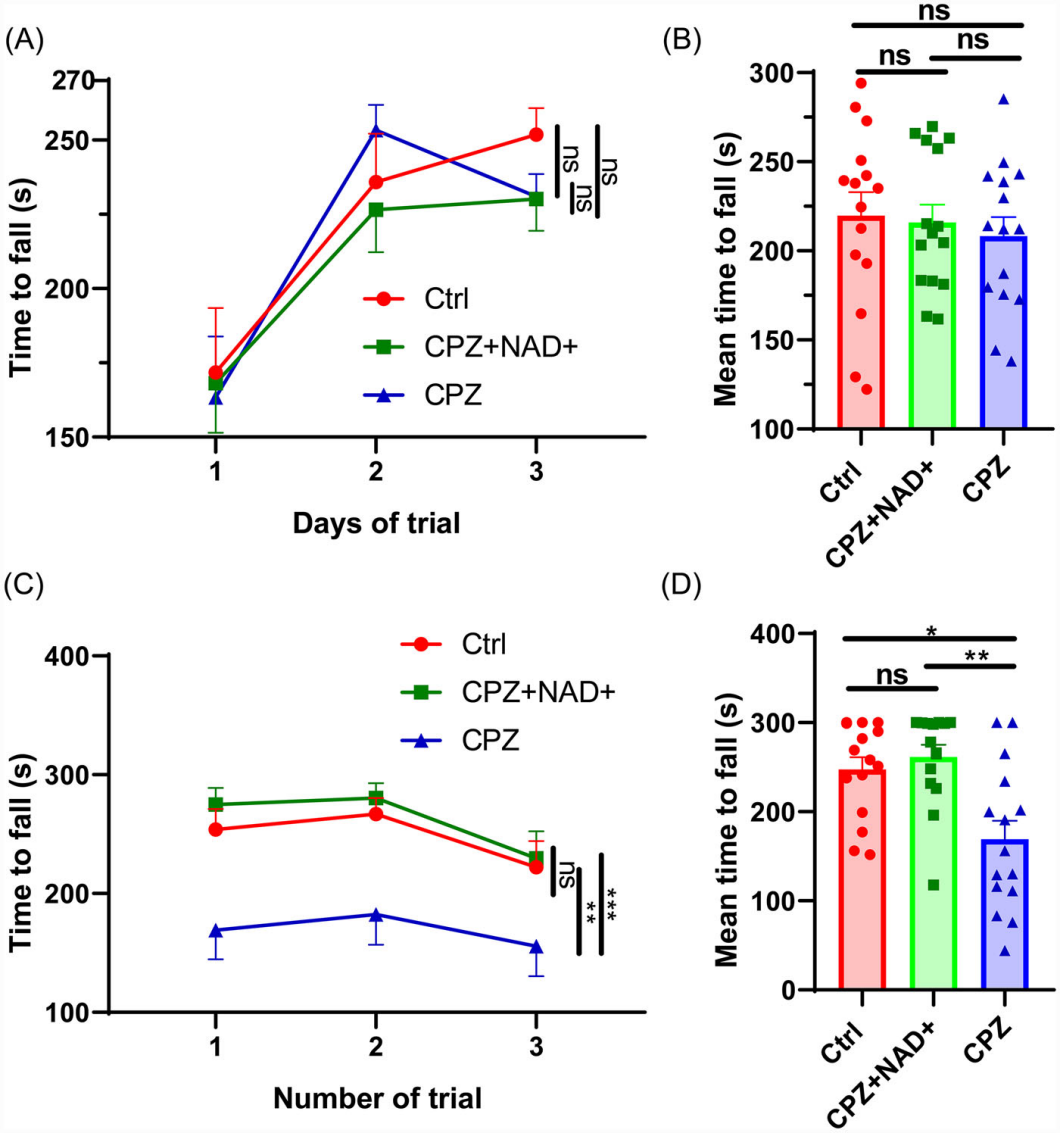
Rotarod test and grip test results of experimental mice. Data were shown as mean ± SEM. *p < 0.05, **p < 0.01, ***p < 0.001, and ns represents not significant. *N* = 15 per group. (A) Time to fall in the Rotarod test among different groups across different experimental days. (B) Mean time to fall among different groups in the Rotarod test. (C) Time to fall in grip test across different experimental trials. (D) Mean time to fall among different groups in grip test.

The time to fall in the Rotarod test of distinct experimental groups showed no significant difference (*p* = 0.759, Figure 3A), and the mean time to fall was also comparable among groups (*p* = 0.768, Figure 3B). However, in the Grip test, the time to fall among groups was significantly different (*p* < 0.001, Figure 3C). The mean time to fall also differed among the groups. The CPZ group showed a reduced mean time to fall (169.10 s), which was significantly lower than that of the Ctrl group (247.60 s, *p* = 0.015) and CPZ+NAD+ group (261.60 s, *p* = 0.003) (Figure 3D).

### 3.3 Emotionality Tests

**FIGURE 4:**
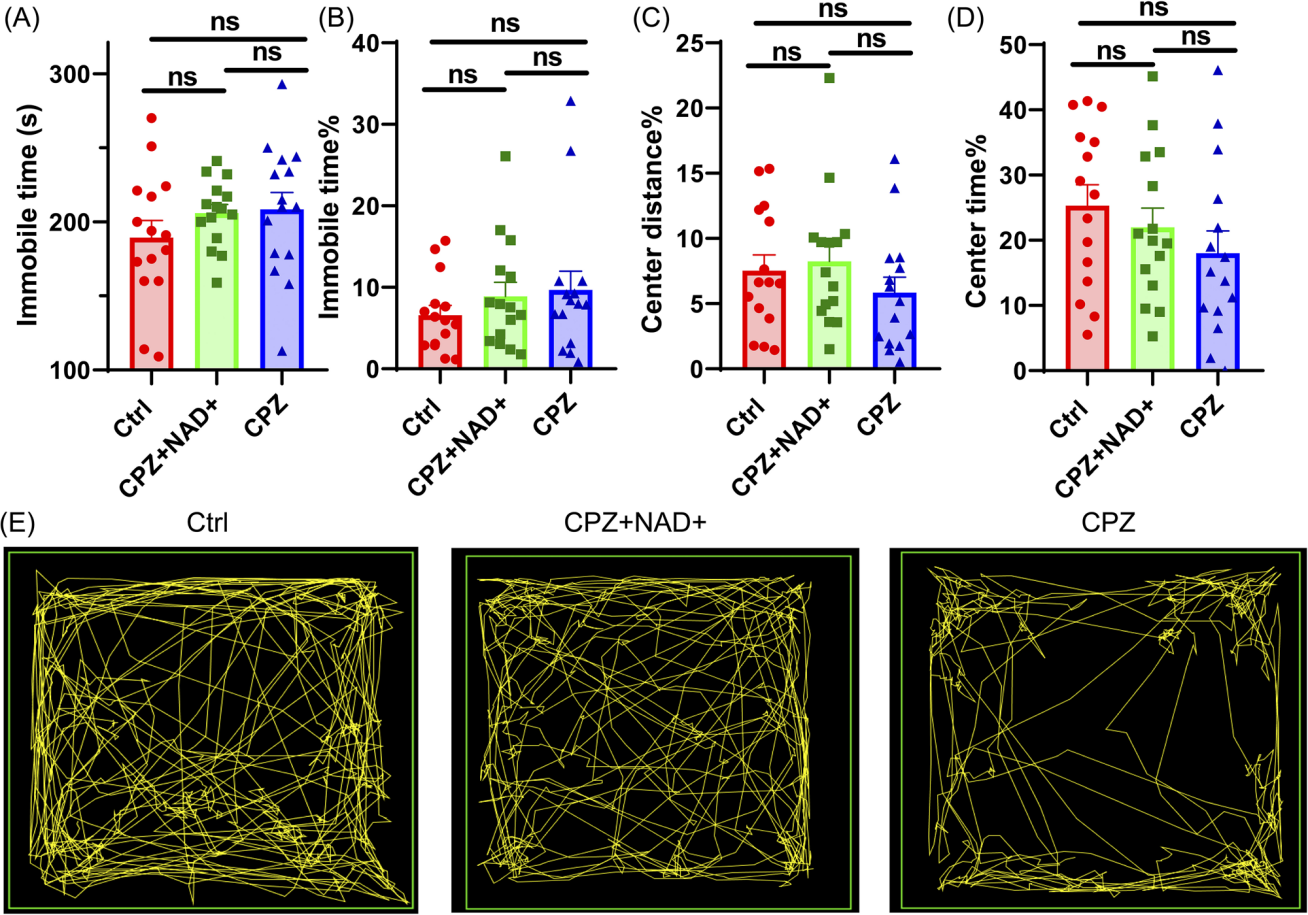
OFT and TST results of experimental mice. Data were expressed as mean ± SEM. *p < 0.05, **p < 0.01, ***p < 0.001, and ns represents not significant. *N* = 15 per group. (A) Immobile time in TST. (B) Immoble time% in OFT. (C) Center distance% in OFT. (D) Center time% in OFT. (E) Typical images of moving tracks. The green square represents the border of the open field, and the yellow line is the moving track of the mice generated by ImageJ software.

The immobile time in the TST test (*p* = 0.349, Figure 4A) and immobile time% in the OFT test (*p* = 0.452, Figure 4B) were comparable among the groups. Center distance% (*p* = 0.388, Figure 4C) and center time% (*p* = 0.281, Figure 4D) also did not show a statistical difference. Representative moving tracks are shown in Figure 4E.

## 4 Discussion

In the present study, to the best of our knowledge, a significant effect of NAD+ supplementation on cuprizone‐induced spatial memory deficiency and locomotor dysfunction was firstly reported.

There have been several pieces of experimental evidence that increasing the intracellular NAD+ level has therapeutic effects on MS‐related animal models. NAD+ supplementation could regulate the T helper cell proportion in the spleen, increase the number of CD11b+ Gr‐1+ myeloid‐derived suppressor cells, increase the expression of immunosuppressive cytokines, such as interleukin 10 and transforming growth factor‐β, and decrease the expression of pro‐inflammatory cytokines, such as interleukin 1β, interleukin 17, and interleukin 18 in the EAE model. The anti‐inflammation and anti‐demyelination effects in the EAE model can be partly attributed to the activation of autophagy, the activation of the adenosine monophosphate‐activated protein kinase/sirtuin 1 pathway, the suppression of the nuclear factor kappa B signaling pathway, and the suppression of nucleotide‐binding oligomerization domain‐like receptor family pyrin domain containing three inflammasomes (Guo et al. 2021; Tullius et al. 2014; J.‐L. Wang et al. 2020; J. Wang et al. 2016; X. Wang et al. 2021). Increased intracellular NAD+ levels ameliorate the demyelination in cuprizone‐induced demyelination mice, and the mechanisms involved are linked to facilitating oligodendrocyte lineage maturation and energy metabolism (Robinson et al. 2020; Roboon et al. 2019). However, whether NAD+ supplementation can ameliorate cuprizone‐induced behavioral dysregulation has not been investigated.

It is possible to increase intracellular NAD+ levels through the uptake of NAD+ precursors, the inhibition of NAD+ degradations, and the promotion of NAD+ synthesis. Supplementation with NAD+ precursors, including but not limited to nicotinic acid, nicotinamide, and nicotinamide mononucleotide, showed a protective effect on many disease conditions (Curran et al. 2025; Qader et al. 2025). However, whether direct NAD+ supplementation can affect the nervous system has been debated due to the following reasons: NAD+ is bioactive and unstable, the extracellular levels of which are much lower than intracellular ones (Jeje et al. 2024; Migaud et al. 2024), and NAD+ can be degraded by ectoenzymes (Bresque et al. 2023). All these factors may prevent supplemented NAD+ from reaching its target organ. However, despite these concerns, low‐dose NAD+ still showed behavioral benefits, suggesting either partial CNS availability or indirect mechanisms.

We speculate that NAD+ supplementation may exert neuroprotective therapeutic effects in the cuprizone mouse model through activating the NAD+ consumer poly(adenosine diphosphate ribose) polymerase for genomic repair (Cohen 2020), and elevating the level of intracellular NAD+ to enhance cell function and activate immune cells via purinergic receptors (Audrito et al. 2021). However, the specific mechanisms still need to be further clarified in future studies.

The majority of MS patients suffer from various behavioral symptoms. It is important to test the effects of NAD+ supplementation on behavioral changes in MS animal models. The MWM test and Y maze test are well‐established behavioral tests used to evaluate the spatial memory of experimental mice. In the present study, after cuprizone intoxication, the spatial memory was impaired, as indicated by decreased target time% and target distance% in the MWM test and decreased alteration ratio in the Y maze test. The direct NAD+ supplementation could increase target time%, target distance%, and alteration ratio, indicating that the intervention has a positive effect on cognitive function in mice.

According to the analysis of the experimental process, the Grip test is more closely related to muscle strength decline in MS patients, and its results better represent muscle strength aspects. The Rotarod test is linked to both muscle strength decline and ataxia in MS patients, and its results reflect motor coordination and muscle strength better. Although the Rotarod experiment yielded negative results, the Grip test revealed that NAD+ supplementation led to significantly improved locomotor ability (more related to muscle strength) in cuprizone‐intoxicated mice. Cuprizone did not lead to a significant decrease in locomotor ability (more related to muscle strength and motor coordination) in the Rotarod test, which contradicts the results of the Grip test. This may be related to the fact that the Rotarod test was conducted earlier than the grip test, possibly resulting in a weaker toxic effect of cuprizone. A potential mechanism for salvaged locomotor ability in the CPZ+NAD+ group is that increased NAD+ levels could enhance the function of mitochondria, which might enhance the function of skeletal muscle for enhancing muscle strength.

A significant portion of MS patients suffer from depression and anxiety (Jellinger 2024), and evidence from animal experiments shows that NAD+ metabolism is disrupted in the condition of anxiety and depression (Chen et al. 2024; Jiang et al. 2025; Xie et al. 2025). The decrease of NAD+ activity is part of the complex changes in the brain of the methylglyoxal‐induced mice depression model (Md et al. 2024), and the NAD+ precursor nicotinamide mononucleotide supplementation by oral gavage improves depressive‐like behaviors by elevating NAD+ biosynthesis and extracellular adenosine triphosphate levels in the medial prefrontal cortex of the chronic social defeat stress‐induced mice depression model (Deng et al. 2024). However, in our experiment, cuprizone and NAD+ supplementation did not cause statistically significant changes in anxiety‐ and depression‐like behaviors in mice. This might be due to the relatively small sample size, the strain‐specific responses of mice, or the fact that anxiety‐ and depression‐like behaviors in the cuprizone‐induced mice demyelination model could not be stably induced (Khalilian et al. 2021; Sen et al. 2019). Whether NAD+ supplementation can improve the anxiety‐ and depression‐like behaviors in mice still needs to be tested in other animal models of anxiety and depression.

To sum up, this study proposes a possibility that cuprizone can lead to decreased motor ability and cognitive impairment in mice, while NAD+ can partially alleviate the symptoms, although the specific mechanism remains unclear. This study highlights the potential of NAD+ as a crucial molecule in the treatment of MS, offering a novel therapeutic direction for addressing cognitive and motor impairments associated with the disease.

This study has several limitations. First, including a group of healthy mice treated with NAD+ would help determine whether NAD+ exerts any enhancing effects on normal physiology or if its benefits are specific to pathological conditions. Second, since MS affects females more frequently, it is necessary for us to include female mice in future experimental plans. Third, due to research funding constraints, we were unable to conduct further mechanistic analyses. In the future, the specific mechanisms by which direct NAD+ supplementation can improve cuprizone‐induced behavioral dysregulation should be systematically studied, including measurement of NAD+ level in the brain after intervention, the pharmacokinetics of direct NAD+ supplementation, multi‐omics analyses of blood, brain, and muscle samples, electrophysiological analyses of brain neural potentials, and pathological examinations of brain tissue, particularly focusing on the hippocampus. Future studies should also investigate whether the effects are mediated by direct central nervous system penetration of NAD+, via its metabolites, or via peripheral actions. These comprehensive studies will offer deeper insights into how NAD+ can modulate neural pathways, ameliorate behavioral abnormalities caused by cuprizone exposure, and potentially impact muscular function. By integrating data from pharmacokinetics, multi‐omics analyses, electrophysiological assessments, and pathological examinations, researchers can gain a more holistic understanding of the effects of NAD+ supplementation and its mechanisms of action in mitigating cuprizone‐induced behavioral dysregulation.

## 5 Conclusion

Direct NAD+ supplementation improves the locomotor ability and spatial memory of cuprizone‐intoxicated male C57BL/6J mice. However, NAD+ supplementation does not show significant effects on depressive and exploratory behavior of experimental mice.

## Author Contributions

**Shuang Song**: formal analysis, investigation, writing – original draft, writing – review and editing. **Ruoyi Guo**: investigation, writing – review and editing. **Jiangyuan Guo**: investigation, writing – review and editing. **Bin Li**: writing – review and editing, conceptualization. **Yusen Han**: investigation, writing – review and editing. **Huining Zhang**: investigation, writing – review and editing. **Li Guo**: conceptualization, writing – review and editing.

## Ethics Statement

The experiment was approved by the Experimental Ethics Committee of the Second Hospital of Hebei Medical University (No. 2021‐AE044).

## Conflicts of Interest

The authors declare no conflicts of interest.

### Peer Review

The peer review history for this article is available at https://publons.com/publon/10.1002/brb3.70525.

## Data Availability

The data presented in this study are available on reasonable request from the corresponding author.
